# Is it possible to differentiate pulmonary sarcoidosis in tumor patients and pulmonary lymphangitic carcinomatosis caused by extrapulmonary tumors on 18F-FDG PET/CT images?

**DOI:** 10.1007/s12672-023-00848-3

**Published:** 2023-12-11

**Authors:** Yu Ji, Yaru Wang, Jie Jiang, Min Chen, Juan Xiao, Zhengjun Dai, Jingsong Zheng

**Affiliations:** 1 Radiology Department, The Second Hospital, Cheeloo College of Medicine, Shandong University, 247 Beiyuan Rd, Jinan, 250033 Shandong China; 2 Radiology Department, Affiliated Hospital of Shandong University of Traditional Chinese Medicine, Jinan, 250011 Shandong China; 3 Internet Medical Department, The Second Hospital, Cheeloo College of Medicine, Shandong University, 247 Beiyuan Rd, Jinan, 250033 Shandong China; 4 Hospital-Acquired Infection Control Department, The Second Hospital, Cheeloo College of Medicine, Shandong University, 247 Beiyuan Rd, Jinan, 250033 Shandong China; 5 Scientific Research Department, Huiying Medical Technology Co., Ltd, Beijing, China; 6 PET/CT Department, Shandong Cancer Hospital and Institute, Shandong First Medical University and Shandong Academy of Medical Sciences, 440 Jiyan Road, Jinan, 250117 Shandong China

**Keywords:** Pulmonary sarcoidosis, Pulmonary lymphangitic carcinomatosis, 18F-FDG, PET/CT, Differential diagnosis

## Abstract

**Purpose:**

Pulmonary sarcoidosis (PS) and pulmonary lymphangitic carcinomatosis (PLC) can be complications in tumor patients, and both involve the pulmonary interstitium and have similar imaging findings. Our objective was to distinguish PS and PLC on 18F-FDG PET/CT images.

**Material and methods:**

The authors reviewed 18F-FDG PET/CT data of PS and PLC, diagnosed based on histopathology and imaging, in patients with tumors from July 2015 to January 2023. Three independent readers performed a blinded comparative analysis of 18F-FDG PET/CT signs in all patients. A multivariate logistic regression model was used to establish a differential diagnosis model.

**Results:**

A total of 114 patients were included in the study: 56 patients with PS (mean age, 56 ± 11 [SD] years; 10 men) and 58 patients with PLC caused by extrapulmonary tumors (mean age, 51 ± 11 [SD] years; 21 men). For PS, breast cancer and cervical cancer were the most common primary tumors. For PLC, breast cancer and gastric cancer were the most common extrapulmonary tumors. The model constructed using multivariate logistic regression consisted of five factors: area of lymph node involvement, bronchovascular bundle diffuse thickening, interlobular septal thickening, pleural effusion, and subpleural hypermetabolic activity. The area under the model characteristic curve was 0.973 (95% CI 0.925–0.994), with a sensitivity, specificity, and positive and negative likelihood ratios of 87.50%, 98.28%, 50.75 and 0.13 respectively.

**Conclusion:**

There are detailed differences in 18F-FDG PET/CT manifestations of PS in tumor patients and PLC caused by extrapulmonary tumors, and the constructed diagnostic model has high clinical application value in differentiating the two.

Since the association between sarcoidosis and malignancy was first reported in the 1974, many studies have suggested that there is a correlation between the occurrence of sarcoidosis and tumors [1–5]. In addition, some antitumor drugs induce a sarcoidosis-like reaction (DISR), characterized by drug-induced systemic granulomas, that is consistent with the clinical, biological, radiological and pathological manifestations of sarcoidosis [6–8]. Especially with the widespread application of immune checkpoint inhibitors, the number of cases of sarcoidosis in patients with tumors has gradually increased [8, 9].

Sarcoidosis mainly involve the lung and thoracic lymph nodes, and approximately 90% of patients present chest radiographic abnormalities at some stage [10, 11]. Pulmonary sarcoidosis (PS) mainly manifests as thoracic lymphadenopathy and granulomas along blood vessels and lymphatic vessels [11–13]. Pulmonary lymphangitic carcinomatosis (PLC) is a pattern of tumor lung metastasis, manifesting as the spread and growth of tumor cells in the pulmonary lymphatic vessels [14, 15]. The anatomical locations and imaging manifestations of PS and PLC are similar. The identification of PS and PLC is closely related to follow-up treatment and the prognosis of patients and has high clinical value. However, some patients are restricted by practice and ethics; it is difficult to obtain pulmonary histopathology, and thus, the diagnosis of PS and PLC via imaging is necessary.

PLC caused by intrapulmonary malignant tumors only invades the hilar lymph nodes on the ipsilateral side of the primary tumor in most cases; contralateral invasion is rare. Even if multiple lobes are involved, interstitial involvement of the lobes where the primary tumor is located is often more severe. Similar results were also found in previous studies on PLC [16–18]. Using this distribution, it is not difficult to distinguish PLC from PS. In our clinical practice, PLC caused by extrapulmonary tumors manifests as bilateral involvement and has a disease distribution and imaging features that are similar to those for PS, so differential diagnosis is more meaningful. Therefore, this study explores the differential diagnosis of PS in tumor patients and PLC caused by extrapulmonary tumors.

At present, the main method to identify PS and PLC is high-resolution computed tomography (HRCT). Previous studies have shown that some morphological signs have significance in distinguishing the two, but there is overlap, and clinical application is limited [14, 19]. In addition, most of the previous studies described the imaging manifestations of PS or PLC as separate diseases, lacking an intuitive comparative study, and there has been no systematic report on the imaging manifestations of PS and PLC caused by extrapulmonary tumors in patient with tumors.

18F-FDG PET/CT is a molecular imaging technique that can reflect glucose metabolism. The vast majority of tumor cells and granulomatous inflammatory lesions exhibit increased glucose uptake and hypermetabolism on PET images [18–22]. The combination of PET/CT with molecular features of the disease undoubtedly introduces new parameters for disease identification. Additionally, with the wide application of PET/CT in tumor diagnosis and treatment, the differentiation of PS and PLC via PET/CT is a challenge that must be addressed. The purpose of this study was to explore 18F-FDG PET/CT imaging differences between PS in tumor patients and PLC caused by extrapulmonary tumors to further identify specific diagnostic signs of the two and to initially construct a differential diagnosis model.

## Materials and methods

This study was approved by our Review Boards for clinical investigation. All of the methods were performed in accordance with the Declaration of Helsinki and the relevant guidelines. Due to the retrospective nature of the study, informed consent was waived.

### Selection of PET/CT Images

We retrospectively analyzed 45823 tumor patients who underwent 18F-FDG PET/CT examination in two hospitals between July 2015 and January 2023. In our image archiving and retrieval system, we used the fuzzy keywords “sarcoidosis”, “pulmonary sarcoidosis”, “pulmonary lymphangitic carcinomatosis”, and “pulmonary interstitial disease” in the first retrieval, and 653 patients were left after the first screening. After the manual exclusion of patients who PLC caused by intrapulmonary tumors or who had incomplete follow-up data. The remaining 209 patients were screened according to the diagnostic criteria for PS and PLC, and ultimately 114 patients were included in the study.

### Scan technique

Imaging of patients was conducted on a PET/CT scanner (TF Big Bore, Philips, Holland; and Ingenuity TF, Philips, Holland; and Biograph Horizon, Siemens, Germany). 18F-FDG with a pH of 5–7 and a radiochemical purity exceeding 95% was produced using a cyclotron (MINItrace, GE Healthcare, Milwaukee WI, USA). The patients underwent fasting for at least 6 h and had a blood glucose levels below 200 mg/dL prior to injection with 18F-FDG. Patients lay in a quiet room 60 min after intravenous injection with 4.4–5.5 MBq/kg 18F-FDG.

Whole-body CT scanning was performed at 120 kVp and 300 mA·s for attenuation correction using a low-dose protocol. PET was performed after Whole-body CT without patient repositioning. PET images were obtained at 7 to 8 couch positions per patient, with an acquisition time of 1.5 min per position. We fused the attenuation-corrected PET and CT images. In order to overcome respiratory interference and better observe lung lesions, chest thin-slice HRCT examinations were performed in neutral breath-hold after PET/CT. Acquisition was performed at 120 kVp with 300 mA·s. Images were reconstructed as contiguous 4 mm slices. Additional lung reformats were generated with contiguous 1 mm slices. Chest thin-slice HRCT was conducted immediately after PET without patient repositioning.

### Reference standard

The biological and imaging features of PS and DISR are similar, and therefore, PS and DISR were classified as PS in this study. The included cases whose pulmonary lesions were newly diagnosed and met the following criteria:

1. Pathological results of lung tissue or mediastinal lymph node biopsy suggested a tumor or noncaseating granuloma,

2. HRCT showed pulmonary interstitial abnormalities, supporting the diagnosis of sarcoidosis or PLC (refer to the study by Criado [23] and Munk [15]),

3. Disease follow-up met the following conditions:

PS: thoracic lesions improved after targeted steroid therapy or lesions improved/changed little in the natural state, PLC: antitumor therapy alleviated thoracic lesions or disease progressed rapidly in the natural state,

The use of steroids as a chemotherapy regimen in the process of anti-tumor treatment is far lower in dosage, frequency, and duration than steroid treatment for sarcoidosis, and does not cause significant changes in sarcoidosis.

The following patients were excluded:

1. Patients with underlying diseases in the lungs (such as pulmonary fibrosis, severe emphysema, and interstitial diseases) or lung structure changes caused by surgery and other reasons,

2. Patients with autoimmune diseases (rheumatic diseases, vasculitis, etc.),

3. Laboratory tests suggest possible infectious lesions in the lungs,

4. Three readers could not agree on imaging features and disease distribution.

### Reading sessions

The images were reviewed by three readers: reader 1 was an attending with 16 year of experience in PET-CT diagnosis; reader 2 was an attending with 12 year of experience in PET-CT diagnosis; and reader 3 was an attending with 25 year of experience in thoracic imaging. They were blinded to the original study interpretation and report, but in knowledge of the clinical tumor. All readers independently analyzed the 18F-FDG-PET/CT images. Because the main purpose of this study was to compare the imaging differences between PS and PLC, to ensure the accuracy of the results, if readers could not agree on imaging features and disease distribution, the case was excluded.

Area of lymph node involvement: Referring to the International Association for the Study of Lung Cancer (IASLC) Lung Cancer Staging Project [24], lymph nodes were divided into 14 stations, namely, 1, 2R, 2L, 3A, 3P, 4R, 4L, 5, 6, 7, 8, 9, 10–11, and 12–14. Additionally, lymph nodes in the 10–11 station were divided into left and right sides, and those in the 12–14 station were subdivided into 5 regions (i.e., 5 lobes). Taking the main location of the involved lymph nodes as the criterion for zoning, each involved zonal lymph node (regardless of the number of lymph nodes) was counted as 1, and the total number was counted. The zonal lymph node count ranged from 0 to 19.

**Fig. 1:**
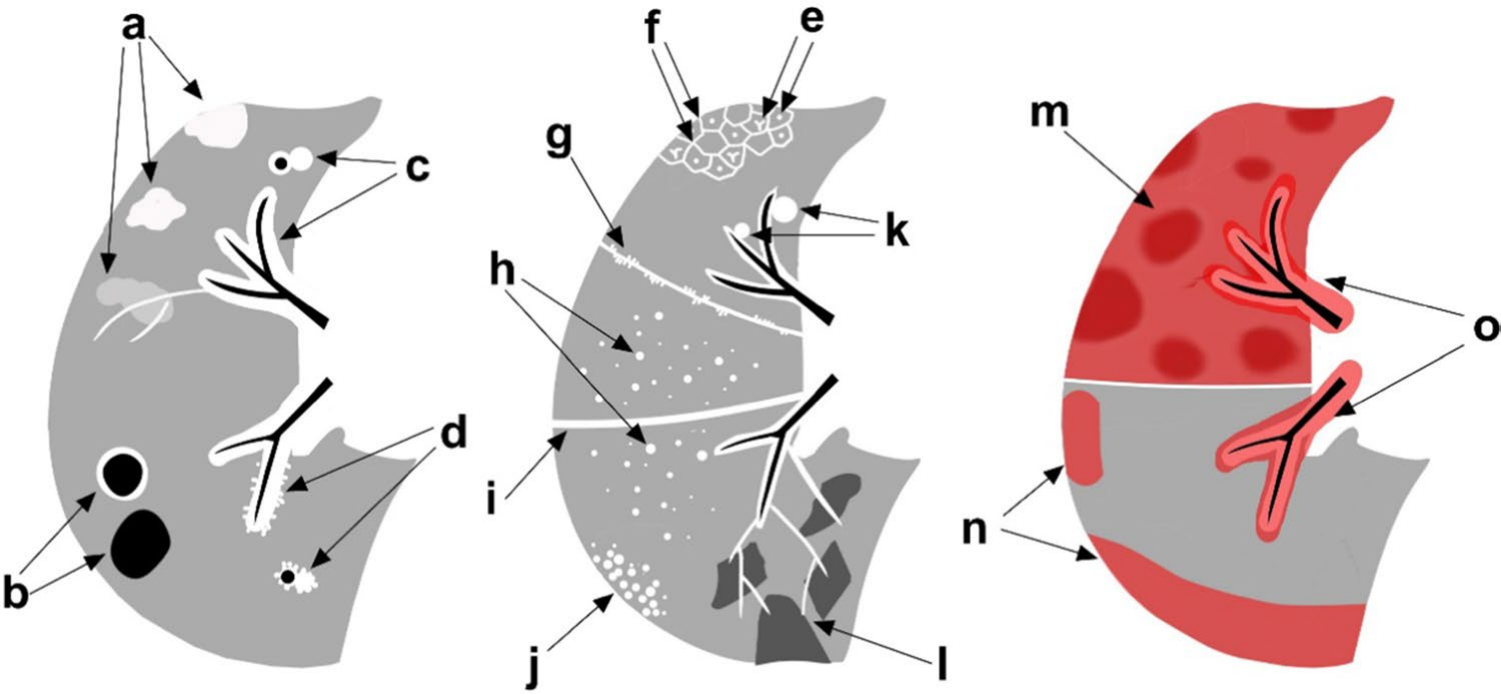
Schematic diagram of pulmonary lesions. a = opacities, b = cysts/cavities, c = bronchovascular bundle diffuse thickening, d = bronchovascular bundle nodular thickening, e = centrilobular peribronchovascular interstitial thickening, f = interlobular septal thickening, g = pleural nodular thickening, h = nodules diffuse distribution, i = pleural diffuse thickening, j = nodules cluster distribution, k = large paravascular solitary nodules, l = mosaic attenuation, m = lobar diffuse hypermetabolism, n = subpleural hypermetabolism, o = bronchovascular bundle hypermetabolism

Lymph node size: The largest lymph node was selected, and its short diameter was measured. When the boundary of the largest lymph node was not clear due to the influence of surrounding tissue, the boundary was delineated with the aid of PET metabolism. The identification of pulmonary nodules was achieved using CT AI-assisted Lung Nodule Detection (version 2.10.0, Huiying Medical Technology Co., Ltd, Beijing, China) for preliminary screening and manual secondary screening to exclude nonnodular interference (such as blood vessel cross-sections and bands of fibrous tissues). Diffuse nodules were defined as the number of pulmonary nodules more than 30 that were randomly distributed. Micronodule were defined as nodules with diameters ≤ 5 mm, and large nodules were defined as nodules with diameters > 5 mm. Dronchovascular bundle nodular/diffuse thickening were defined as nodular/smooth thickening of the interstitial tissue in the centrilobular region manifests as nodular/smooth thickening of the bronchi or pulmonary arteries. Subpleural hypermetabolic activity were defined as Various forms of hypermetabolic lesions appear under the pleura, exhibiting significantly higher metabolic activity compared to the lung background. Figure 1 provides the definitions for all of the characteristics of intrapulmonary lesions.

### Follow-up

All patients received at least one FDG PET/CT or chest CT follow-up within 2 months after the diagnosis of PS or PLC. All patients in this study had histopathological evidence (lung tissue or lymph nodes) at the time of enrollment. Due to ethical limitations, some patients did not undergo lung parenchymal pathology and only obtained histopathological results from lymph node samples. Therefore, the main purpose of follow-up is to ensure accurate diagnosis. 1–2 cycles of anti-tumor or anti-sarcoidosis therapy can effectively reflect changes in the course of the disease. The follow-up time of this study is based on the needs of tumor follow-up and does not impose additional radiation exposure or costs on the patients.

### Statistical analysis

The software package R (v3.5.3, https://www.r-project.org/) was used for statistical analysis. For continuous variables, means and standard deviations or medians with interquartile ranges were calculated. For categorical variables, absolute numbers with percentages were recorded. The independent samples t-test or Mann—Whitney U test was used to compare quantitative data. Pearson’s chi-square tests or Fisher’s exact tests, where appropriate, were used to compare the difference in qualitative data.

Variables that are significant at a level of 0.05 in the univariate analysis were included in the multivariate logistic regression analyses to build a radiological diagnostic model. A bidirectional stepwise elimination approach was used to simplify the model on the basis of the Akaike information criterion. The variance inflation factor was used to measure multicollinearity, and a value 0.10 was a criterion for judging the multicollinearity of factors. Receiver operating characteristic (ROC) curves were constructed to evaluate the diagnostic performance of variables.

All tests were 2-sided, and a P value < 0.05 was considered statistically significant; confidence intervals for proportions are reported as 2-sided exact binomial 95% CIs.

## Results

### Demographic and clinical data

A total of 114 patients were included in the study: 56 tumor patients with pulmonary sarcoidosis and 58 patients with PLC caused by extrapulmonary tumors. There were 10 males and 46 females in the PS group (age, 55.9 ± 10.6 years; range, 36–87 years old); the PLC group included 21 males and 37 females (age, 51.3 ± 10.7 years; range, 31–90 years). Out of the total cases, 21 patients were diagnosed through lung tissue biopsy, including 14 cases of PS and 7 cases of PLC. The remaining 93 cases underwent lymph node biopsy combined with follow-up for diagnosis. In the PS group, breast cancer and cervical cancer were the most common primary tumors. Breast cancer and gastric cancer were the most common extrapulmonary tumors in the PLC group. The clinical data are provided in Table 1.

**Table 1:** Demographic and Baseline Clinical Characteristics of the Participants

Variable	PS in tumor patients (n = 56) No. (%)	PLC caused by extrapulmonary tumors (n = 58) No. (%)
Primary tumor^†^		
	Breast cancer (19, 33.9%)	Breast cancer (17, 29.3%)
	Cervical cancer (8, 14.3%)	Gastric cancer (11, 19.0%)
	Thyroid cancer (4, 7.1%)	Cervical cancer (7, 12.1%)
	Esophageal carcinoma (4, 7.1%)	Unknown primary focus (7, 12.1%)
	Lymphoma (4, 7.1%)	Colon cancer (4, 6.9%)
	Rectal cancer (3, 5.4%)	Liver cancer (2, 3.4%)
	Lung cancer (3, 5.4%)	Bile duct carcinoma (2, 3.4%)
	Gastric cancer (2, 3.6%)	Duodenal cancer (2, 3.4%)
	Oropharynx cancer (1, 1.8%)	Rectal cancer (2, 3.4%)
	Adrenocortical carcinoma (1, 1.8%)	Renal carcinoma (1, 1.7%)
	Sarcoma (1, 1.8%)	thymic carcinoma (1, 1.7%)
	Renal carcinoma (1, 1.8%)	Parotid gland carcinoma (1, 1.7%)
	Melanoma (1, 1.8%)	Ovarian cancer (1, 1.7%)
	Bile duct carcinoma (1, 1.8%)	
	Pancreatic cancer (1, 1.8%)	
	Colon cancer (1, 1.8%)	
	Endometrial cancer (1, 1.8%)	

Unless otherwise specified, data are numbers of participants, with percentages in parentheses. *PS*  pulmonary sarcoidosis. *PLC* pulmonary lymphangitic carcinomatosis

^†^ The numbers of participants and percentages in parentheses

### Lymph node features of PS and PLC

The lymph node characteristics and semiquantitative parameters of PS and PLC are shown in Table 2. The area of lymph node involvement, the incidence of bilateral hilar involvement, the short diameter of lymph nodes, and the average SUVmax were higher in the PS group than those in the PLC group, and the differences were significant (P < 0.01). There was no difference in the occurrence of calcified lymph nodes.

**Table 2:** Comparison the Lymph Node Features Between PS and PLC

Variable	PS in tumor patients (n = 56) No. (%)	PLC caused by extrapulmonary tumors (n = 58) No (%)	P value
Area of lymph node involvement^*^	12 (10, 13)	8 (5, 10)	** < 0.01**
Station of lymph node involvement			** < 0.01**
1	34 (60.7%)	42 (72.4%)	
2R	49 (87.5%)	26 (44.8%)	
2 L	20 (35.7%)	11 (19.0%)	
3A	30 (53.6%)	21 (36.2%)	
3P	6 (10.1%)	1 (1.7%)	
4R	56 (100.0%)	45 (77.6%)	
4 L	49 (87.5%)	32 (55.2%)	
5	50 (89.3%)	32 (55.2%)	
6	32 (57.1%)	13 (22.4%)	
7	52 (92.9%)	42 (72.4%)	
8	42 (75.0%)	20 (34.5%)	
9	17 (30.4%)	2 (3.4%)	
10–11^†^	110 (98.2%)	88 (75.9%)	
12–14^††^	204 (72.6%)	82 (28.3%)	
Hilar lymph node involvement			** < 0.01**
No involvement	1 (1.8%)	7 (12.1%)	
Unilateral hilar involvement	0	14 (24.1%)	
Bilateral hilar involvement	55 (98.2%)	37 (64.8%)	
Range	4–15	0–15	
Calcification	4 (7.1%)	5(8.6%)	1.000
Short diameter, mm^**^	17.9(5.6)	13.1(5.5)	** < 0.01**
SUVmax^**^	15.1(7.5)	8.4 (5.1)	** < 0.01**
Range	3.9–33.6	1.2–28.7	

Unless otherwise specified, data are numbers of participants, with percentages in parentheses. *PS*  pulmonary sarcoidosis, *PLC* = pulmonary lymphangitic carcinomatosis, *SUVmax*  maximum standardized uptake value

^*^Data is reported as the median, with the quartile in parentheses

^**^Data is reported as the mean, with the standard deviation in parentheses

^†^ The lymph node count in the 10–11 region ranged from 0 to 2 (depending on the number of hilar involvement)

^††^ The lymph node count in the 12–14 region ranged from 0 to 5 (depending on the number of lobe involvement)

### Pulmonary features of PS and PLC

**Fig. 2:**
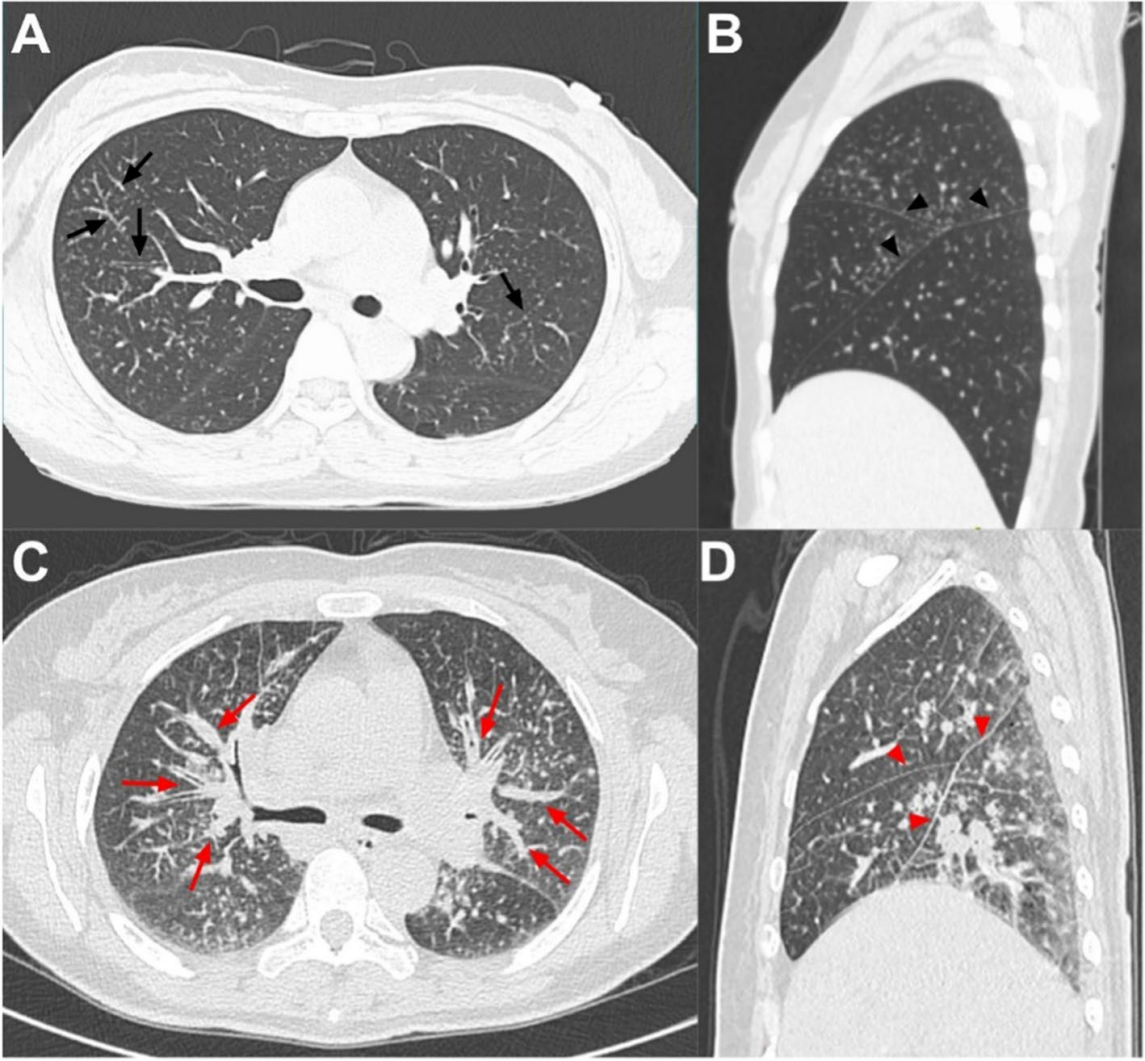
**A**, **B**, Axial CT and sagittal CT image in a 46-year-old endometrial cancer woman with pulmonary sarcoidosis showed bronchovascular bundle (black arrows) and pleural nodular thickening (black triangle). **C**, **D**, Axial CT and sagittal CT image in a 54-year-old ovarian cancer woman with pulmonary lymphangitic carcinomatosis showed bronchovascular bundle (red arrows) and pleural diffuse thickening (red triangle)

**Fig. 3:**
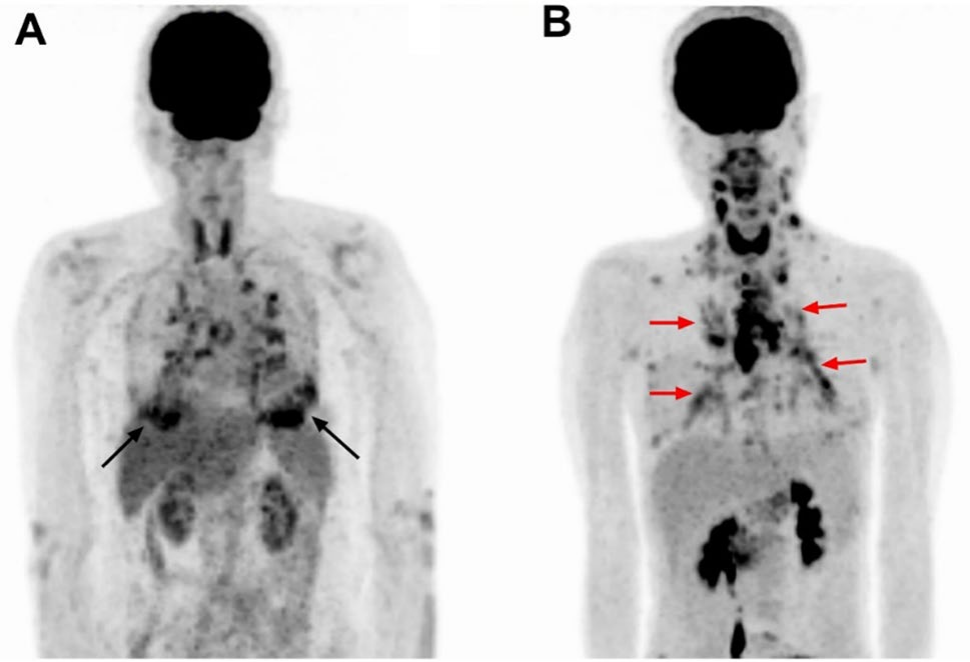
A, Maximum intensity projection 18F-FDG PET/CT image in a 63-year-old breast cancer woman with pulmonary sarcoidosis showed intense tracer uptake in the peripheral area of both lungs (black arrows). B, Maximum intensity projection 18F-FDG PET/CT image in a 50-year-old colon cancer woman with pulmonary lymphangitic carcinomatosis showed intense tracer uptake in the central area of both lungs (black arrows)

**Fig. 4:**
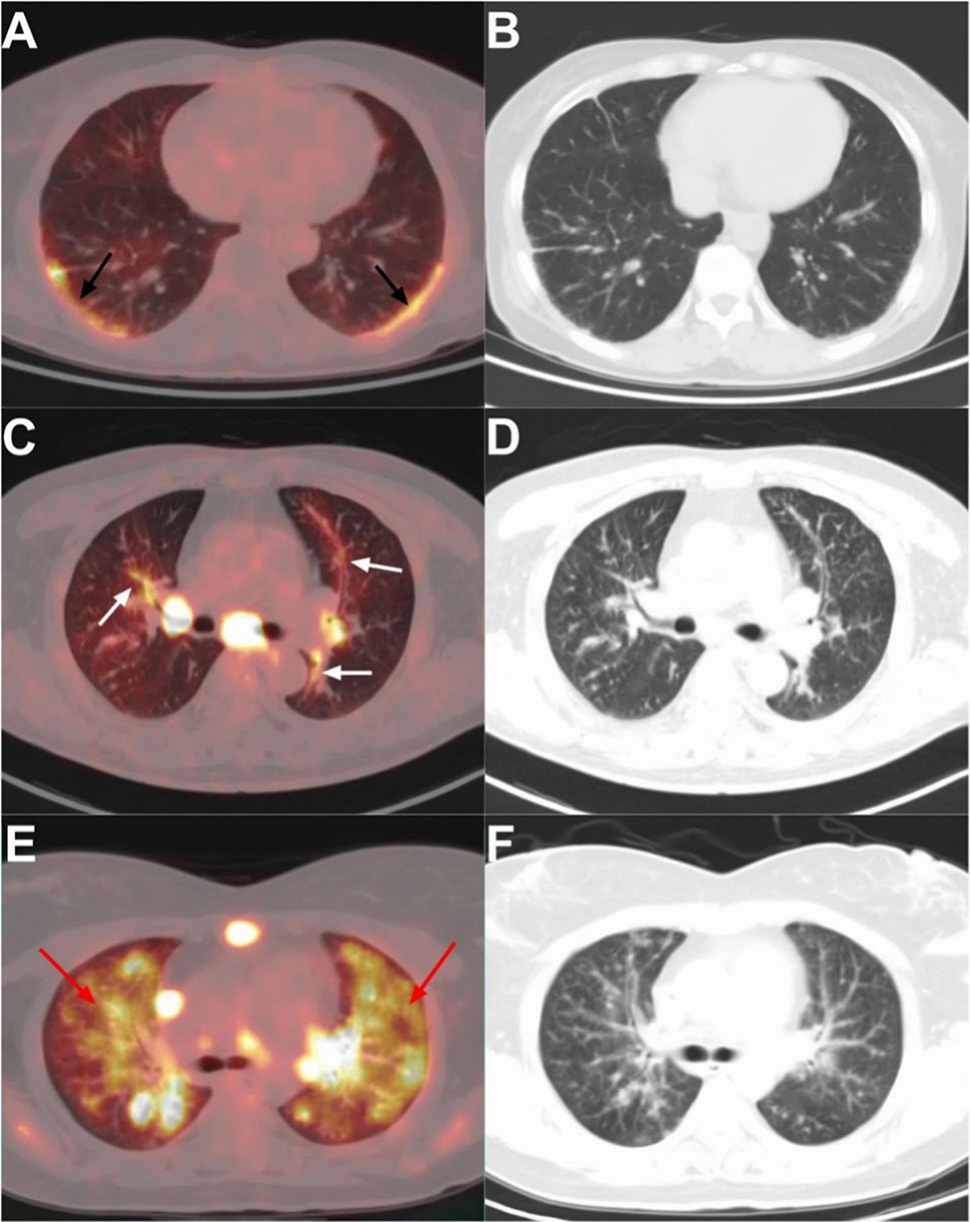
**A**, **B**, Axial fused 18F-FDG PET/CT image in a 55-year-old pancreatic cancer woman with pulmonary sarcoidosis showed subpleural hypermetabolism (black arrows; SUVmax, 5.3), and low-dose axial CT showed subpleural micronodules and pseudopleural plaques. **C**, **D**, Axial fused 18F-FDG PET/CT image in a 57-year-old gastric cancer man with pulmonary lymphangitic carcinomatosis showed Bronchovascular bundle hypermetabolism (white arrows; SUVmax, 4.0), and low-dose axial CT showed Bronchovascular bundle thickening. **E**, **F**, Axial fused 18F-FDG PET/CT image in a 31-year-old colon cancer man with pulmonary lymphangitic carcinomatosis showed lobar diffuse hypermetabolism (red arrows; SUVmax, 8.1), and low-dose axial CT showed multiple nodules and opacities in the lungs

The characteristics of intrapulmonary lesions and semiquantitative parameters of PS and PLC are shown in Table 3. The incidence of bronchovascular bundle thickening, pleural thickening, interlobular septal thickening, centrilobular peribronchovascular interstitial thickening, and pleural effusion in was lower in the PS group than in the PLC group; while the number of involved pulmonary lobes, the incidence of large paravascular solitary nodules and average SUVmax of intrapulmonary lesions in PS group was higher than those in PLC group (P < 0.05). There was no significant difference between the two groups in nodule size and distribution, intrapulmonary opacity, cyst/cavity, and mosaic attenuation. Regarding the interstitial thickening pattern, nodular thickening was predominant in the PS group, and diffuse thickening was predominant in the PLC group (Fig. 2). In terms of metabolic pattern, subpleural hypermetabolic activity was predominant in the PS group, and bronchovascular bundle hypermetabolic activity and lobar diffuse hypermetabolic activity were more common in the PLC group (Fig. 3, 4).

**Table 3:** Comparison the Pulmonary Features Between PS and PLC

Variable	PS in tumor patients(n = 56) No. (%)	Extrapulmonary tumor caused PLC (n = 58) No (%)	P value
Extent of lung involvement			0.105
Unilateral involvement	12 (21.4%)	6 (10.3%)	
Bilateral involvement	44 (78.6%)	52 (89.7%)	
Number of lobes involved^*^	4 (2, 5)	4 (4, 5)	** < 0.01**
Position of lobar involvement			** < 0.01**
Right upper lobe	41 (73.2%)	48 (82.8%)	
Right middle lobe	36 (64.3%)	46 (76.3%)	
Right lower lobe	44 (78.6%)	55 (94.8%)	
Left upper lobe	39 (69.6%)	42 (72.4%)	
Left lower lobe	35 (62.5%)	47 (81.0%)	
Bronchovascular bundle			** < 0.01**
No thickening	38 (67.9%)	10 (17.2%)	
Nodular thickening	10 (17.9%)	3 (5.2%)	
Diffuse thickening	8 (14.3%)	45 (77.6%)	
Pleural			** < 0.01**
No thickening	17 (30.4%)	6 (10.3%)	
Nodular thickening	30 (53.6%)	14 (24.1%)	
Diffuse thickening	9 (28.3%)	38 (65.5%)	
Interlobular septal thickening	20 (35.7%)	57 (98.3%)	** < 0.01**
Centrilobular peribronchovascular interstitial thickening	2 (3.6%)	30 (51.7%)	** < 0.01**
Micronodules	52 (92.9%)	56 (96.6%)	0.434
Large nodule	40 (71.4%)	42 (72.4%)	0.907
Nodules diffuse distribution	24 (42.9%)	29 (50.0%)	0.445
Nodules cluster distribution	7 (12.5%)	2 (3.4%)	0.059
Large paravascular solitary nodule	18 (32.1%)	5 (8.6%)	** < 0.01**
Opacity	33 (58.9%)	35 (60.3%)	0.878
Cyst/cavity	8 (14.3%)	7 (12.1%)	0.726
Mosaic attenuation	6 (10.7%)	3 (5.2%)	0.453
Pleural effusion	4 (7.1%)	33 (56.9%)	** < 0.01**
Pulmonary SUV_max_^**^	6.6 (3.9)	5.2 (4.0)	0.023
Subpleural hypermetabolism	29 (51.8%)	9 (15.5%)	** < 0.01**
Bronchovascular bundle hypermetabolism	19 (33.9%)	43 (74.1%)	** < 0.01**
lobar diffuse hypermetabolism	7 (12.5%)	26 (44.8%)	** < 0.01**

Unless otherwise specified, data are numbers of participants, with percentages in parentheses. *PS*  pulmonary sarcoidosis, *PLC* pulmonary lymphangitic carcinomatosis, *SUVmax*  maximum standardized uptake value

^*^Data is reported as the median, with the quartile in parentheses

^**^Data is reported as the mean, with the standard deviation in parentheses

### Establishment of the multivariate model

**Fig. 5:**
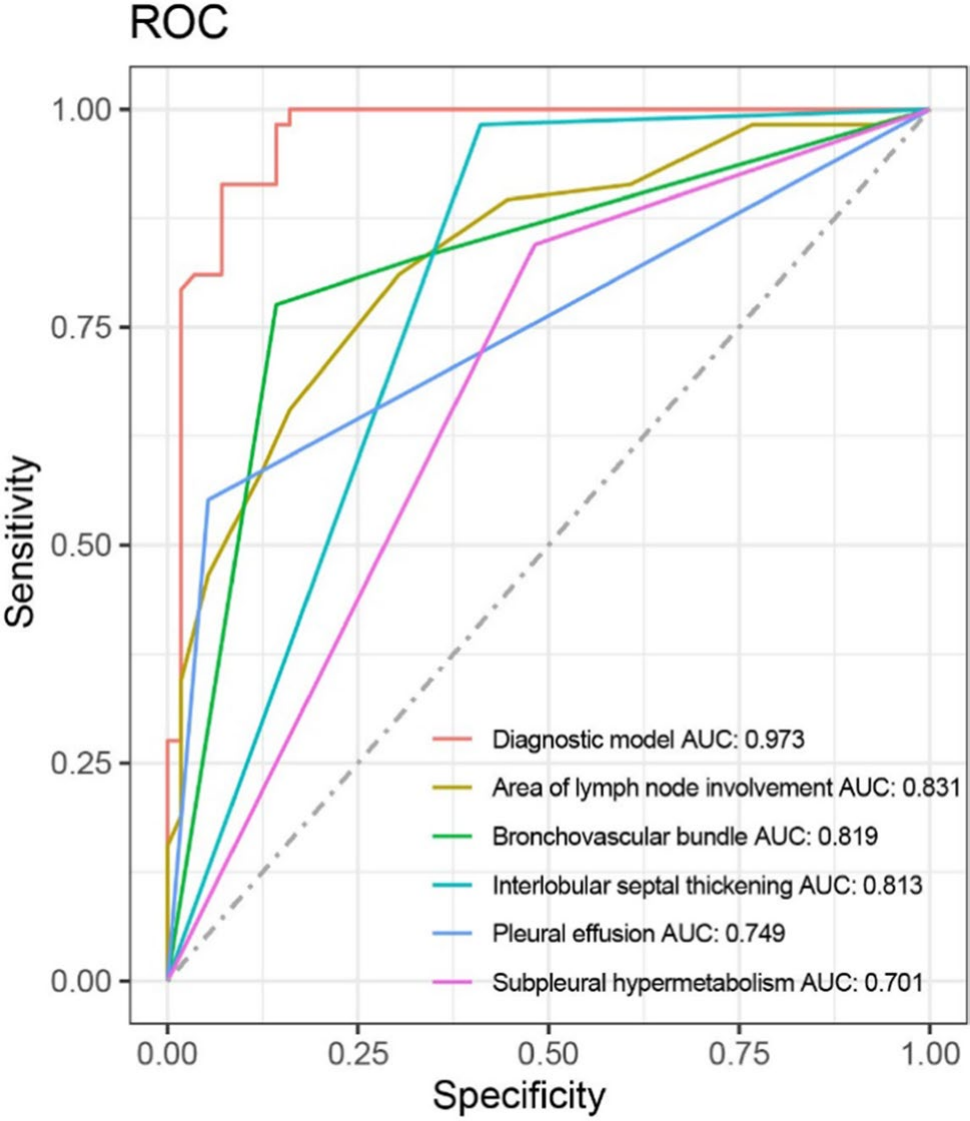
Graphs show results of receiver operating characteristic (ROC) curve analysis of diagnostic model and univariate variables for distinguishing between pulmonary sarcoidosis in tumor patients and pulmonary lymphangitic carcinomatosis caused by extrapulmonary tumors. *AUC*  area under the ROC curve

The diagnostic performance of this model was then assessed. Based on the stability and fitting effect of the model, five imaging features, i.e., area of lymph node involvement, bronchovascular bundle diffuse thickening, interlobular septal thickening, pleural effusion and subpleural hypermetabolic activity, were selected to establish a radiological diagnosis model (Table 4). The area under the curve (AUC) in differentiating PS in tumor patients and PLC caused by extrapulmonary tumors was 0.973 (95% CI 0.925–0.994), with a sensitivity, specificity, and positive and negative likelihood ratios of 87.50%, 98.28%, 50.75 and 0.13 respectively (Fig. 5).

**Table 4:** Multivariate Model of 18F-FDG PET/CT Features Associated With PS/PLC

Variable	β	Odds ratio (95% CI)	P value
Area of lymph node involvement	0.619	0.440–0.870	0.006
Bronchovascular bundle			
No thickening	Reference	Reference	
Nodular thickening	0.601	0.052–6.954	0.684
Diffuse thickening	9.234	1.552–54.950	0.015
Interlobular septal thickening			
No thickening	Reference	Reference	
Thickening	23.975	2.356–243.938	0.007
Pleural effusion			
No effusion	Reference	Reference	
Effusion	9.881	1.206–80.970	0.033
Subpleural hypermetabolism			
No hypermetabolism	Reference	Reference	
Hypermetabolism	0.037	0.005–0.304	0.002

## Discussion

In this study, we constructed a new differential diagnosis model by combining disease distribution, radiological characteristics, and metabolic patterns, considering clinical significance and model stability. Five PET/CT features were included in this model, namely, area of lymph node involvement, bronchovascular bundle diffuse thickening, interlobular septal thickening, pleural effusion, and subpleural hypermetabolic activity. The application of this model has good performance in the differentiation of PS in tumor patients and PLC caused by extrapulmonary tumors (AUC is 0.972), serving as an effective guide in clinical practice.

We assessed the lymph nodes involved in PS and PLC and found that the area of lymph node involvement in the PS group was greater than that in the PLC group, suggesting that sarcoidosis has a wider range of lymph node involvement. We believe that this difference is because the involved lymph nodes in sarcoidosis are usually multicentric in origin whereas the involved lymph nodes in PLC are caused by tumor metastasis and follow the path of lymph node metastasis; therefore, they are smaller in extent. Both the short diameter and SUVmax of lymph nodes in PS are larger than those in PLC, but in practice, these two indicators have a high degree of overlap; therefore, using these indicators alone is of little value for identification.

PET/CT has high value in judging whether lymph nodes are involved. The high sensitivity of PET/CT can accurately identify lymph nodes (especially level 12–14) that are not large and difficult to identify by CT alone, thus greatly enhancing the diagnostic confidence of clinicians. In this study, calcifications in both PS and PLC were rare, with no significant difference between groups, a finding that is inconsistent with other studies showing that sarcoidosis is prone to lymph node calcification [25, 26]. We believe that this difference in results is due to the disease course: the PS patients included in our study all had a history of tumors and were followed up frequently, and the time for lymph node calcification was not reached when PS was diagnosed. The study by Miller et al. found that the incidence of calcification in PS patients was 3% after 5 years and 20% after 10 years, results that confirm our findings [27]. Therefore, it is difficult to use “lymph node calcification” to differentiate PS and PLC in patients with tumors.

In this study, both PS and PLC were common bilaterally, but PLC involved more lobes and more significant interstitial changes. The phenomenon of bilateral lung involvement in PLC suggests that the tumor first metastasizes to the lungs, then invades the adjacent lung lymphatic system, and then spreads in the lymphatic system, leading to hilar and mediastinal lymph node metastasis. In this study, 7 patients had only pulmonary interstitial involvement without lymph node metastasis, a finding that supports the speculation. Similar findings have been found in previous autopsies [28, 29]. Therefore, PLC caused by extrapulmonary tumors has a wide extent and is serious. PLC with interstitial edema mostly show diffuse interstitial thickening, and nodular changes are not prominent. PS granulomas are located outside the lymphatic vessels, and the possibility of severe edema caused by the compression of the lymphatic vessels is not high; therefore, PS mostly manifests as interstitial nodular thickening [13, 30]. “Bronchovascular bundles and pleural thickening” is often used to describe the interstitial changes in the two diseases, but the results of our study showed that differences in interstitial thickening patterns are more useful for differential diagnosis.

We found that nodules were common in PS and PLC, with no difference in nodule size and distribution pattern. However, the large paravascular solitary nodules was more common in PS patients than in PLC patients (32.1% vs. 8.6%). Although this sign can occur in both diseases, the mechanism is different: the former involves the hyperplasia of interstitial granulomas around the great vessels, and the latter involves hematogenous metastases in the lungs randomly located next to the great vessels. All or most large nodules in the lung located next to blood vessels is strongly suggestive of PS. This differential sign was first proposed in this study, although it is not highly sensitive, it has strong specificity and is of great significance in the identification of the two diseases.

Previous literature has considered mosaic attenuation and pulmonary fibrosis specific signs of PS and of great significance in the diagnosis of PS [13, 14, 23]. However, in this study, the incidence of these signs was low and no differential significance. We also believe that it is the cause of the early stage of the disease. This finding also reflects the characteristics of PS in patients with tumors; that is, the disease is often in the early stage, and some specific signs that appear in the late stage are difficult to be used to distinguish between PS and PLC.

Although the mean SUVmax of intrapulmonary lesions in PS was higher than that in PLC, there was a large overlap between them, thus offering limited value in clinical differentiation. At this point, metabolic pattern has more clinical value. Three metabolic distribution patterns were investigated in this study, namely, bronchovascular bundle hypermetabolic activity (central lung area), subpleural hypermetabolic activity (peripheral lung area), and lobar diffuse hypermetabolic activity. The results showed that PS mostly exhibited subpleural metabolic activity, and that PLC mainly increased metabolism in the bronchovascular bundles and diffuse lobes. PS predominantly involves the extrapulmonary zone, and subpleural hypermetabolic sites are often accompanied by micronodule, patchy ground-glass opacity or consolidation shadows. Intense 18F-FDG uptake and no airspace filling in the corresponding parts are more likely to be formed by the fusion of granulation in the lung interstitium and alveolar cavity rather than simple alveolitis. The pathological results reported by Nishimura et al. also support this conclusion [13]. PLC exhibited more hypermetabolism in the axial interstitium or diffuse lobar hypermetabolism, and these manifestations were less common in PS, reflecting the presence of diffuse infiltration and proliferation of tumor cells in the axial interstitium, peripheral interstitium, and intralobular interstitium, as found in previous autopsy results [15, 31]. However, the extent of 18F-FDG hypermetabolism in some patients was significantly lower than the abnormal range of CT and even showed diffuse interstitial involvement without hypermetabolism; that is, CT and PET did not match. We believe that there are two possibilities for this manifestation: one is that the tumor blocks the proximal lymphatic vessels, resulting in interstitial edema, and there are fewer tumor cells in the interstitium; the other is that a few special tumors (such as mucinous adenocarcinoma, etc.) uptake 18F-FDG less. The high sensitivity of 18F-FDG PET/CT can more intuitively reflect the overall distribution of PS and PLC and can better differentiate the two from MIP images.

This study has several limitations. First, the data were retrospective, and the constructed discriminative model needs to be further validated using external data. Second, although the initial number of patients in this study was large, the inclusion and exclusion criteria were strict, and the number of valid samples in each group was small, potentially leading to some bias in the study results.

## Conclusion

PS in tumor patients and PLC caused by extrapulmonary tumors mainly invade the pulmonary interstitium and lymph nodes, but there are differences in distribution and PET/CT signs between the two, ccurate differentiation of the two has significant clinical value in managing tumor patients. Tumor patients with PS typically exhibit early-stage PS manifestations, which have relatively specific characteristics. Moreover, single imaging signs may overlap in 18F-FDG PET/CT, making it difficult to effectively differentiate diagnosis. Therefore, in clinical practice, it is necessary to integrate anatomical and metabolic features, combine metabolic values with metabolic patterns, and comprehensively consider multiple imaging findings in order to achieve accurate differentiation diagnosis. In this study, a diagnostic model constructed with five parameters, “lymph node involvement area”, “bronchovascular bundle diffuse thickening”, “interlobular septal thickening”, “pleural effusion” and “subpleural hypermetabolic activity”, was used to differentiate PS in tumor patients and PLC caused by extrapulmonary tumors and has high clinical application value.

## Data Availability

The datasets generated during and/or analysed during the current study are available from the corresponding author on reasonable request.

## Abbreviations

FDG: Fluorodeoxyglucose
SUVmax: Maximum standardized uptake value
PS: Pulmonary sarcoidosis
PLC: Pulmonary lymphangitic carcinomatosis
